# Night-time radiative warming using the atmosphere

**DOI:** 10.1038/s41377-023-01315-y

**Published:** 2023-11-10

**Authors:** Yining Zhu, Yiwei Zhou, Bing Qin, Rui Qin, Min Qiu, Qiang Li

**Affiliations:** 1 State Key Laboratory of Modern Optical Instrumentation, College of Optical Science and Engineering, Zhejiang University, Hangzhou, 310027 China; 2 Key Laboratory of 3D Micro/Nano Fabrication and Characterization of Zhejiang Province, School of Engineering, Westlake University, Hangzhou, 310024 China; 3 Institute of Advanced Technology, Westlake Institute for Advanced Study, Hangzhou, 310024 China

**Keywords:** Optical materials and structures, Mid-infrared photonics

## Abstract

Night-time warming is vital for human production and daily life. Conventional methods like active heaters are energy-intensive, while passive insulating films possess restrictions regarding space consumption and the lack of heat gain. In this work, a nanophotonic-based night-time warming strategy that passively inhibits thermal radiation of objects while actively harnessing that of atmosphere is proposed. By using a photonic-engineered thin film that exhibits high reflectivity (~0.91) in the atmospheric transparent band (8–14 μm) and high absorptivity (~0.7) in the atmospheric radiative band (5–8 and 14–16 μm), temperature rise of 2.1 °C/4.4 °C compared to typical low-e film and broadband absorber is achieved. Moreover, net heat loss as low as 9 W m^−2^ is experimentally observed, compared to 16 and 39 W m^−2^ for low-e film and broadband absorber, respectively. This strategy suggests an innovative way for sustainable warming, thus contributes to addressing the challenges of climate change and promoting global carbon neutrality.

## Introduction

Warming, as a basic part of human production and life, plays a crucial role in many industrial and agricultural processes. While solar irradiance provides most of the Earth’s energy during the day, night-time warming is even more challengeable due to its absence. At night, the temperature near the Earth’s ground can drop to sub-freezing point due to direct radiative transfer towards outer space (~3 K) through the atmospheric transparent band^1,2^ (8–14 μm). For example, desert regions exhibit an average night-time temperature of −3.9 °C in stark contrast to 38 °C during noon-time^3^, leading huge threat to various industries. For agriculture, excessively low temperature can damage the living plants and thus reduce the crop yield^4,5^. For outdoor transportation, frost and ice can form on the power lines, posing safety hazards to energy supply^6–9^. Therefore, warming at night-time has emerged as a critical issue in many areas.

Achieving night-time warming has conventionally relied on active approaches such as electrically driven heaters, which come with several drawbacks, including the need for additional bulky power supply systems, high energy consumption, and increased carbon emissions. Passive methods typically involve the use of insulating blankets or low emissivity (low-e) film to reduce heat loss^10–12^. For insulating blankets, conduction and convection are impeded by the presence of a certain volume of space^13,14^; however, their highly emissive surfaces can still cause heat loss. Low-e films, on the other hand, can reduce radiation loss from the object^15,16^ but simultaneously block all radiative heat gains from external environment. Consequently, advanced low-carbon and efficient method for night-time warming with straightforward implementation has so far slipped out of research at status quo.

In this work, we propose a nanophotonic-based night-time warming strategy which passively inhibit thermal radiation of the object while actively utilizing that of atmosphere. The atmosphere, with high specific heat capacity, can hold a relatively higher temperature than the Earth’s ground at night^17^, thus serving as an external heat source for ground object. The strategy is implemented by covering the object with a photonic-engineered thin film, which exhibits a high reflectivity of 0.91 in the atmospheric transparent band (8–14 μm), and high absorptivity of 0.7 in the atmospheric radiative band (5–8 and 14–16 μm). Outdoor night-time experiments reveal that, through this selective reflection/absorption control, the temperature of the covered object can increase by 2.1 °C/4.4 °C compared to broadband reflector (BR, namely low-e film) and broadband absorber (BA), respectively. Furthermore, net heat loss as low as only 9 W m^−2^ is observed in the experiment, in contrast to 16 W m^−2^ and 39 W m^−2^ for broadband reflector and broadband absorber, respectively. We believe that this novel night-time warming strategy suggests new avenues for sustainable warming, thus offering considerable potential in addressing challenges of climate change and promoting carbon neutrality.

## Results

### Theoretical analysis

**Fig. 1:**
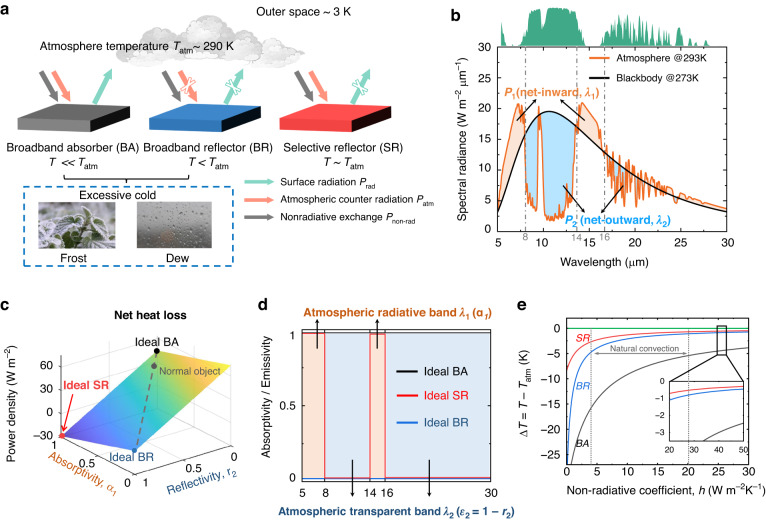
Working mechanisms of night-time hybrid radiative warming with a selective reflector/absorber. **a** Schematic diagram of the radiative heat exchange among the atmosphere, the outer space, and the surfaces with different optical properties. **b** Spectral radiance of atmosphere at 293 K and object (assumed as blackbody) at 273 K. The orange area denotes the net-inward power *P*_1_ in atmospheric radiative band, and the light blue area denotes the net-outward power *P*_2_ in atmospheric transparent band. The green area above denotes the atmospheric transmittance. **c** Net heat radiative loss of surfaces with different *α*_1_ and *r*_2_, which are the absorptivity and reflectivity in atmospheric radiative band *λ*_1_ and atmospheric transparent band *λ*_2_, respectively. The atmosphere and surface temperature are set to be 293 K and 278 K, respectively. **d** Spectral absorptivity / emissivity of the broadband absorber (BA), broadband reflector (BR), and selective reflector (SR). **e** Simulated temperature difference at thermal equilibrium between atmosphere (293 K) and surfaces with different radiative properties as a function of non-radiative coefficient, *h*

To numerically estimate the heat transfer process among the atmosphere, the outer space and the object with various optical properties, a one-dimensional steady state model is employed^18–20^ (Fig. 1a). The net heat loss of an object with temperature *T* is given by:

1 $${P}_{{\rm{net}}}={P}_{{\rm{rad}}}(T)-{P}_{{\rm{atm}}}({T}_{{\rm{atm}}})-{P}_{{\rm{non}}-{\rm{rad}}}$$

${P}_{{\rm{rad}}}(T)$ and ${P}_{{\rm{atm}}}({T}_{{\rm{atm}}})$ is the thermal radiation emitted by the surface and the atmosphere, which can be calculated by Planck’s equation. ${P}_{{\rm{non}}-{\rm{rad}}}=h({T}_{\rm{atm}}-T)$ is the non-radiative heat exchange including convection and conduction, where $h$ is the non-radiation coefficient.

To comprehensively explore the contribution of wavelength-dependent absorptivity/reflectivity to radiative warming, atmospheric radiative band (5–8 and 14–16 μm, *λ*_1_) and atmospheric transparent band (8–14 and 16 μm+, *λ*_2_) are considered separately based on the atmospheric transmittance^21–24^ (Fig. 1b). The atmosphere, when holding a higher temperature than the object, exhibits stronger thermal radiation in the *λ*_1_ band, primarily resulting from water vapor emission^25,26^. Conversely, in the *λ*_2_ band, the atmospheric radiation is weaker, as the atmosphere is nearly transparent in this band. The net heat loss thus can be written as:

2 $${P}_{{\rm{net}}}={\alpha }_{1}(T){P}_{1}+{\varepsilon }_{2}(T){P}_{2}-{P}_{{\rm{non}}-{\rm{rad}}}$$

where ${P}_{1}$ and ${P}_{2}$ are the net radiative fluxes between the object and the atmosphere in atmospheric radiative band *λ*_1_ and atmospheric transparent band *λ*_2_, respectively (Fig. 1b). Defining net-outward radiative flux as positive, ${P}_{1}$ is negative, indicating the radiative heat gain of the atmosphere to the object in *λ*_1_ band, while ${P}_{2}$ is positive, representing the radiative heat loss of the object in *λ*_2_ band. ${\alpha }_{1}(T)$ and ${\varepsilon }_{2}(T)$ are the temperature-dependent averaged absorptivity and reflectivity of the object in atmospheric radiative band and atmospheric transparent band, respectively (See “Materials and methods” for details).

The net radiative heat loss *P*_net_ varying with *α*_1_ and *r*_2_ can be calculated (Fig. 1c). An ideal photonic-engineered device (defined as “ideal selective reflector (SR)”) for night-time warming should possess high absorptivity in the atmospheric radiative band (*α*_1_ = 1) to maximize heat gain, and high reflectivity in the atmospheric transparent band (*r*_2_ = 1) to minimize radiative loss. For comparison, most objects that act as broadband absorbers have high net heat losses, for example, blackbody has a net heat loss of up to 50 W m^-2^. While for broadband reflector, the high reflectivity significantly reduces the net heat loss, but the low absorptivity limits the possibility of introducing extra heat gain. The ideal reflector has zero heat loss, but the ideal selective reflector can have a net heat gain of up to 30 W m^-2^.

Considering the impact of non-radiative processes, the equilibrium temperature of the SR is always higher than that of BA and BR for different non-radiation coefficients, *h* (Fig. 1e). Notably, when non-radiative heat transfer is eliminated (*h* ≈ 0), the SR’s temperature experiences a mere 8 °C drop, while the BA and BR’s temperature decrease by over 32 °C with respect to atmospheric temperature (*T*_atm_). In the case of natural convection (3 < *h* < 20), the SR exhibits averaged temperature rises of 1.1 °C/7.8 °C compared to BR/BA, respectively. Even under conditions where external convection dominates (*h* > 20), the selective reflector still outperforms broadband absorber and broadband reflector (inset in Fig. 1e).

### Design and characterization

**Fig. 2:**
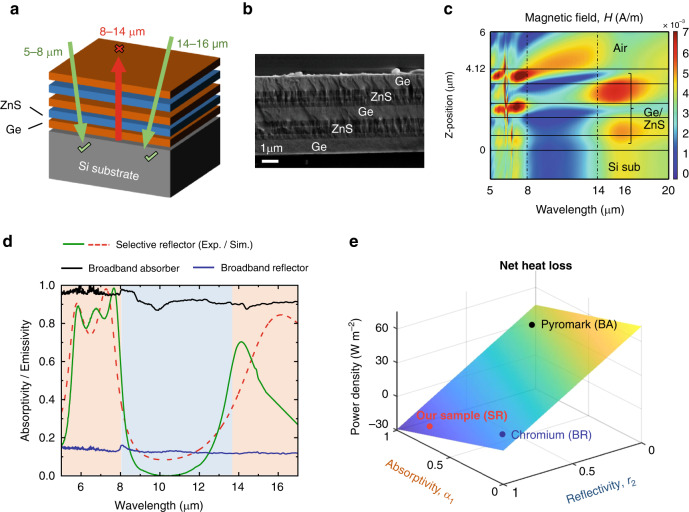
Design and fabrication of the selective reflector. **a** Designed Ge/ZnS multilayer film on a silicon substrate. **b** SEM image of the multilayer film cross section. **c** Calculated magnetic field intensity inside and at the interface of the multilayer film for normal plane wave incidence. **d** Measured absorptivity / emissivity of the selective reflector (green), the broadband absorber (black), and the broadband reflector (blue). The simulated absorptivity of the selective reflector (red dashed line) is also presented. **e** Calculated net radiative loss of our sample (SR), Pyromark (BA), and Chromium (BR) based on the measured spectra

Based on the analysis above, we design and fabricate a photonic film-based selective reflector (Fig. 2). The whole structure comprises two parts: a germanium/zinc sulfide multilayer film and an absorptive substrate. The Ge/ZnS stacks (see Fig. S1 for refractive index) form a distributed Bragg reflector in the atmospheric transparent band (8–14 μm), rendering the remaining atmospheric radiative band (5–8 and 14–16 μm) to be transparent (Fig. 2a). Therefore, atmospheric radiation can be transmitted and absorbed by the substrate. Through careful tuning of the thickness of each layer guided by an optimization algorithm (Method), a selective reflector consisted of merely five layers of Ge/ZnS with a total thickness of 4.12 μm is achieved on a silicon wafer. Figure 2b shows the scanning electron microscope cross-section of the multilayers (see “Materials and methods” for detailed fabrication). The calculated magnetic field intensity inside and at the interface of the multilayer film is presented in Fig. 2c. For wavelengths of 5–8 and 14–16 μm in the atmospheric radiative band, high transmittance is achieved, enabling the substrate to effectively absorb heat from the atmospheric radiation. For wavelengths of 8–14 μm in the atmospheric transparent band, thermal radiation from the substrate is blocked, as illustrated by the decaying magnetic field.

The spectral absorptivity of the selective reflector is compared with typical broadband absorber and broadband reflector (see Method for materials), as illustrated in Fig. 2d. For the atmospheric transparent band (8–14 μm), the average emissivity of SR is 0.09, corresponding to a high reflectivity of 0.91 (*r*_2_), which is higher than that of BA (0.1) and BR (0.88). For the atmospheric radiative band (5–8 and 14–16 μm), the average absorptivity (*α*_1_) of the SR is 0.7, higher than that of the BR (0.13). Based on the measured spectra, the net radiative heat exchange of different surfaces are numerically estimated, as illustrated in Fig. 2e. The fabricated selective reflector can reach a net heat gain of 14 W m^−2^, while in contrast, the broadband reflector and the broadband absorber both have net heat loss of 5 W m^−2^ and 40 W m^−2^, respectively.

### Night-time warming performance

**Fig. 3:**
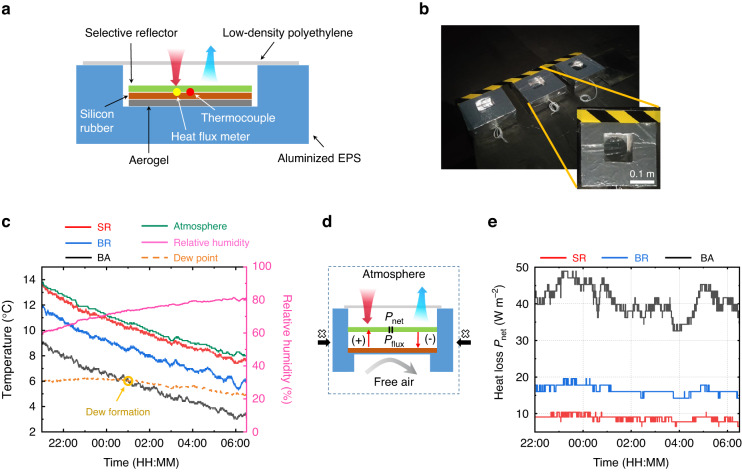
Experiment of night-time warming performance of the selective reflector. **a** Schematic diagram and **b** photograph of the experimental setup. **c** Real-time temperature of selective reflector (red), broadband reflector (blue), broadband absorber (black), and atmosphere (green). The relative humidity (pink, right y-axis) is also recorded, and the dew point (yellow dashed line) is calculated with it. **d** Schematic illustration of measuring heat loss through a heat flux meter. ‘+’ or ‘-’ means the direction of the heat flow is outward or inward. **e** Measured real-time heat loss of surfaces with different optical properties

The night-time warming performance of the selective reflector is experimentally evaluated in an outdoor environment on a clear winter night when warming is greatly needed. As depicted in the longitudinal section diagram of the setup in Fig. 3a, the selective reflector is placed on a silicone rubber, surrounded by an aluminized expanded polystyrene box, and shielded with low-density polyethylene to minimize non-radiative heat exchange effects^27,28^. A thermocouple is placed between the selective reflector and the substrate to record real-time surface temperature. For comparison, a broadband absorber and a broadband reflector are put in identical test boxes and situated side by side on a rooftop (Fig. 3b, see details in “Materials and methods”).

As illustrated in Fig. 3c, the average temperature of the SR during the test is 4.4 °C and 2.1 °C higher than that of the BA and BR, respectively, indicating the remarkable warming performance of the SR. Moreover, the temperature of the SR keeps close to the atmosphere temperature (*T*_SR_ ≈ *T*_atm_) throughout the entire night, which is attributed to the efficient heat exchange between the SR and the atmosphere through the atmospheric radiative band. This temperature similarity enables the SR to prevent dew formation (*T*_SR_ > *T*_dew_) even under high relative humidity conditions, since the dew point is always less than or equal to the atmospheric temperature. Conversely, for BA and BR, the surface temperatures are more likely to fall below the dew point, resulting in unwanted condensation or freezing. Dew formation occurs at around 1 a.m. for BA and persists through the night (Fig. 3c). Experiments with direct exposure to air also reveal a temperature rise of 1.1 °C between SR and BA (Fig. S2). Additionally, tests on a cloudy night indicates that the SR outperforms BA and BR under both cloudy and cloudless conditions (Fig. S3).

The net heat loss (*P*_net_) is also assessed and compared using a heat flux meter (Fig. 3d). To maintain adjacent temperature for the three groups, free air is introduced by allowing ventilation beneath the sample (Fig. 3d). As depicted in Fig. 3e, the averaged net heat loss of SR, BR, and BA are 9.1, 16.0, 39.8 W m^−2^, respectively. The reduced heat loss of the SR indicates that suppressing thermal radiation in atmospheric transparent band and absorbing heat from atmospheric radiation synergistically contribute to night-time warming.

### Anti-condensation potential

**Fig. 4:**
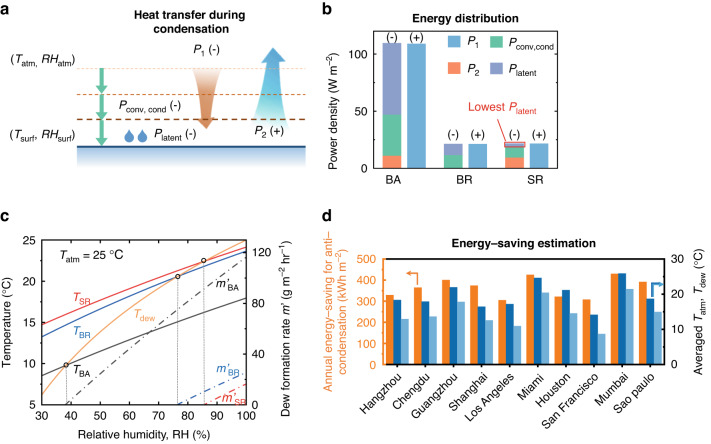
Modeled reduction of dew formation and energy-saving estimation. **a** Schematic diagram of the heat exchange modes at the interface of a surface where condensation is occurring. ‘+’ or ‘-’ means that the direction of the heat flow is outward or inward. **b** The power density distribution of different heat transfer modes for BA, BR, and SR. **c** Calculated surface temperature (solid line, left y-axis) and dew formation rate (dash dot line, right y-axis) as a function of relative humidity for BA (black), BR (blue), and SR (red). **d** Calculated annual energy-saving for anti-condensation (left y-axis), averaged atmosphere temperature and dew point (right y-axis) of 10 representative cities in various climate zones

Condensation on surfaces such as power lines and wind turbines can pose significant safety hazards to industrial operations. Various passive anti-condensation methods have been widely studied, encompassing super-hydrophobic surfaces^29^, anti-icing surfaces^30^, and solar-assisted heating^31^. However, each comes with certain limitations. Here, we numerically estimate the prospective applications of the nanophotonic engineered selective reflector for anti-condensation. The heat exchange processes at the interface of a surface where condensation occurs are illustrated in Fig. 4a. From the atmosphere to the surface, the air temperature gradually decreases while the relative humidity correspondingly increases. When condensation occurs, the relative humidity of the air above the surface (*RH*_surf_) reaches 100%, and the dew formation rate $m\text{'}$ is proportional to the latent heat released by condensation process, which can be written as: $m\text{'}=\frac{{P}_{{\rm{latent}}}}{{\varDelta }_{{\rm{latent}}}}$. ${\varDelta }_{{\rm{latent}}}$ is the latent heat of water per unit mass. ${P}_{{\rm{latent}}}$ can be calculated by combining Eq. (2) with mass transfer equation^32,33^ (see Supplement 1 for details). Through calculating the power density of different heat transfer modes for BA, BR, and SR at the condensation state, we get an energy distribution diagram, shown in Fig. 4b. Due to inhibited radiation in atmospheric transparent band and enhanced absorption in atmospheric radiative band, the SR exhibits the lowest *P*_latent_ compared to BA and BR, which corresponds the slowest dew formation rate.

The RH dependence of surface temperature and dew formation rate for surfaces with varying optical properties is also calculated (Fig. 4c). As the relative humidity increases, the condensation occurs when surface temperature is lower than the dew point (orange line in Fig. 4c), known as the critical relative humidity point, *RH*_critical_. For BA, BR, and SR, the *RH*_critical_ values are 38%, 76%, and 86%, respectively, indicating that the SR is the least susceptible to condensation. The annual reduction of energy-saving for anti-condensation is calculated in 10 representative cities with distinct temperature and dew point profiles (Fig. 4d). The calculation is conducted by comparing the heating power required to maintain the temperature above the dew point with and without the selective reflector, based on the hourly night-time temperature and relative humidity data of each city throughout 2022 (see Supplement 2 for details). Remarkably, the selective reflector can bring about an energy-saving effect of approximately 400 kWh per square meter, which is more pronounced in humid cities where the difference between the average atmosphere temperature and dew point is minimal.

## Discussion

To conclude, we have presented a night-time warming strategy incorporating a photonic-engineered thin film that passively inhibits thermal radiation of objects while actively harnessing that of atmosphere. First, through night-time outdoor test, we experimentally demonstrate a temperature rise of 2.1 °C/4.4 °C compared to low-e film and broadband absorber, respectively. Second, our selective reflector reduces the net heat loss to only 9 W m^-2^. It is conceivable that by designing nanophotonic structures^34–42^ with a more closely matched spectrum to the ideal selective reflector, or conducting test in more extreme environments, a larger net heat gain up to 60 W m^-2^ could be attained (Fig. S4). Moreover, the proposed multilayer film can be fabricated on various substrates using a scalable, low-cost roll-to-roll manner, facilitating potential applications such as sustainable thermal management^43–48^, anti-freezing^6,29,31^, anti-fogging^49,50^, and energy generation^2,51–59^. Last, the passive and straightforward implementation method offers the opportunity to enable electricity-free warming in off-grid areas, including rural agricultural fields. Ultimately, the innovative photonic strategy for night-time warming heralds a promising approach to energy conservation in diverse scenarios, thereby paving the way for a new paradigm in the pursuit of carbon neutrality.

## Materials and methods

### Net heat loss calculation

The absorptivity and reflectivity in λ_1_ and λ_2_ bands are given by:

3 $${\alpha }_{1}(T)=\frac{\int d{\lambda }_{1}\alpha (\lambda ){I}_{{\rm{BB}}}(\lambda ,T)}{\int d{\lambda }_{1}{I}_{{\rm{BB}}}(\lambda ,T)}$$

4 $${r}_{2}(T)=1-\frac{\int d{\lambda }_{2}\varepsilon (\lambda ){I}_{{\rm{BB}}}(\lambda ,T)}{\int d{\lambda }_{2}{I}_{{\rm{BB}}}(\lambda ,T)}$$

where $${\lambda }_{1}\in [5\,{\rm{\mu }}{\rm{m}},8\,{\rm{\mu }}{\rm{m}}]\cup [14\,{\rm{\mu }}{\rm{m}},16\,{\rm{\mu }}{\rm{m}}]$$ and $${\lambda }_{2}\in [8\,{\rm{\mu }}{\rm{m}},14\,{\rm{\mu }}{\rm{m}}]\cup [16\,{\rm{\mu }}{\rm{m}},30\,{\rm{\mu }}{\rm{m}}]$$.

${I}_{{\rm{BB}}}(\lambda ,T)=\frac{2h{c}^{2}}{{\lambda }^{5}}\frac{1}{{e}^{hc/(\lambda {k}_{B}T)-1}}$ is the spectral radiance of a blackbody at temperature *T*, where *h* is Planck’s constant, and *k*_*B*_ is Boltzmann constant. The net radiative fluxes *P*_1_ and *P*_2_ can be calculated as:

5 $$\begin{array}{ll}{P}_{1,2}=A\int d\varOmega \,\cos \theta \Big[\int d{\lambda }_{1,2}{I}_{{\rm{BB}}}(\lambda ,T)\\\qquad-\,\int d{\lambda }_{1,2}{I}_{{\rm{BB}}}(\lambda ,{T}_{{\rm{atm}}}){\varepsilon }_{{\rm{atm}}}(\lambda )\Big]\end{array}$$

where $\int d\varOmega =2\pi {\int }_{0}^{\pi /2}d\theta$ is the angular integral ${\varepsilon }_{{\rm{atm}}}(\lambda )$ is the hemispherical emissivity of the atmosphere, given by

6 $${\varepsilon }_{{\rm{atm}}}(\lambda )={\int }_{0}^{\pi /2}d\theta \,\sin \theta \,\cos \theta \left[1-t{(\lambda )}^{1/\cos \theta }\right]$$

where *t*(λ) is the atmospheric transmittance in the zenith angle.

### Film design and optimization

The target metrics chosen for optimization is to maximize reflectance in atmospheric transparent band, and ideal transmittance in atmospheric radiative band. A periodic structure is pre-generated in which Ge (*n*_1_ = 4) and ZnS (*n*_1_ = 2.2) are arranged alternatively to match the band equation of one-dimensional photonic crystal: ${\omega }_{{\rm{c}}}=\frac{\pi c}{{n}_{1}{d}_{1}+{n}_{2}{d}_{2}}$, where *ω*_c_ is the central frequency of the band, d_1_ and d_2_ are the thickness. A genetic algorithm is used to re-optimize the thicknesses and reduce the layer numbers.

### Sample fabrication and preparation

For selective reflector, the Ge/ZnS multilayer film is deposited with E-beam evaporation on a silica substrate, with deposition rates of 0.5 nm/s (Ge) and 1.5 nm/s (ZnS). The thickness of each layer from top to bottom are Ge 747 nm, ZnS 908 nm, Ge 712 nm, ZnS 1006 nm, and Ge 765 nm, respectively. For broadband absorber, a 0.3 mm thick black tape is attached to a silica substrate. For broadband reflector, a 100 nm thick layer of chromium is deposited on a silica substrate through magnetron sputtering. Before preparation, the silica substrates are ultrasonic cleaned with acetone and deionized water.

### Optical measurements

MIR absorptivity/emissivity is measured with a Fourier transform infrared spectrometer (Bruker, Vertex 70) with a room-temperature doped triglycine sulfate (DTGS) detector. Black soot deposited on a gold-coated silicon wafer using a burning candle is used as a reference.

### Thermal measurement

The apparatus containing the selective reflector is a 30 cm × 30 cm × 12 cm foam box (expanded polystyrene) hollowed out in the center of the top surface. The size of the hollow part is 11 cm × 11 cm × 6 cm. Inside the chamber, a piece of 1-cm-thick silica aerogel is placed on the bottom, on which a piece of 5-mm-thick silicone rubber covered with the selective reflector is placed. The aerogel, the rubber, and the emitter with same sizes of 10 cm × 10 cm are held together with clamps. A 12-μm-thick low-density polyethylene film is used to shield the wind and transmit the infrared radiation. A 2D heat analysis of the setup is performed in COMSOL to better understand the non-radiative effect in the experiment (Fig. S5). The temperature measurements are performed with thermocouples (Omega, SA-1K), and the heat flux measurements are performed with a heat flux meter (FHF02, Hukseflux). A recorder (KSB24A0R) is used to acquire the real-time temperature and heat flux. Both thermocouples and the heat flux meter are attached under the sample. An instrument shelter is used to record real-time atmosphere temperature, relative humidity, and wind speed.

### Supplementary information

Supplementary information for Night-time Radiative Warming Using the Atmosphere
