# Supplementary material for: Night-time radiative warming using the atmosphere
Source: Light Sci Appl. 2023 Nov 10;12:268. doi: 10.1038/s41377-023-01315-y (PMC10638402; doi:10.1038/s41377-023-01315-y)
Supplement: Supplementary file 1 — Supplementary information for Night-time Radiative Warming Using the Atmosphere [file 41377_2023_1315_MOESM1_ESM.docx]

Supplementary information for

**Night-time Radiative Warming Using the Atmosphere**

*Yining Zhu^1^, Yiwei Zhou^1^, Bing Qin^1^, Rui Qin^1^, Min Qiu^2,3^, and Qiang Li^1,*^*

^1^State Key Laboratory of Modern Optical Instrumentation, College of Optical Science and Engineering, Zhejiang University, Hangzhou 310027, China

^2^Key Laboratory of 3D Micro/Nano Fabrication and Characterization of Zhejiang Province, School of Engineering, Westlake University, Hangzhou 310024, China

^3^Institute of Advanced Technology, Westlake Institute for Advanced Study, Hangzhou 310024, China

E-mail: qiangli@zju.edu.cn

**Supplement 1 Thermal analysis of a flat surface during condensation**

Here, the radiation exchanges in atmospheric radiative band (λ_1_) and atmospheric transparent band (λ_2_) are considered separately to explore the contribution of wavelength-dependent absorptivity / reflectivity.

The energy balance at surface where condensation occurs can be written as

$-P_{\text{latent}}+\alpha_{1}(T_{surf})P_{1}+(1-r_{2}(T_{surf}))P_{2}-P_{\text{conv,cond}}=0$ (S1)

where $P_{\text{latent}}$is the latent heat released by condensation process, $P_{1}$ and $P_{2}$ are the net radiative fluxes between the object and the atmosphere in atmospheric radiative band λ_1_ and atmospheric transparent band λ_2_, respectively. (See Method for detailed calculation)

The dew formation rate $m'$ can be derived from two conditions: 1) energy flow and 2) mass transfer, respectively. The energy flow equation can be written as

$m'=\frac{P_{\text{latent}}}{\Delta_{\text{latent}}}$ (S2)

The mass transfer equation can be written as:

$m'=g_{m.H_{2}O}(\hat{m}_{H_{2}O,atm}-\hat{m}_{H_{2}O,surface})$ (S3)

where $g_{m.H_{2}O}$ is the mass transfer coefficient of water, $\hat{m}_{H_{2}O,atm}$ and $\hat{m}_{H_{2}O,surface}$ are the mass fraction of water vapor in the atmosphere and near the surface, repectively.

The dew formation rate then can be calculated by combining the above three equations. All parameters are calculated with the equations (3-6, S4-S7) and Table S1

Expressions of terms involved in heat transfer analysis

$P_{\text{conv,cond}}=h(T_{atm}-T_{surf})$ (S4)

$\hat{m}_{H_{2}O}=\frac{RH\times P_{H_{2}O}M_{H_{2}O}}{P_{\infty}M_{air}}$ (S5)

$g_{m.H_{2}O}=\frac{h}{c_{p}}\left(Le\right)^{n}$ (S6)

$P_{H_{2}O}=133.32*10^{[a-b/(T+c)]}$ (S7)

**Table S1** Parameters used in the analysis of heat transfer model.

| Symbol | Definition | Value | Unit |
| --- | --- | --- | --- |
| $\Delta_{\text{latent}}$ | Latent heat of water condensation per unit mass | 2260 | [kJ kg^-1^] |
| $\alpha_{1}$ | Averaged absorptivity in atmospheric radiative band | Selective reflector: 0.7  Broadband absorber: 0.9  Broadband reflector: 0.13 | Unitless |
| $r_{2}$ | Averaged reflectivity in atmospheric radiative band | Selective reflector: 0.91  Broadband absorber: 0.1  Broadband reflector: 0.88 | Unitless |
| *RH* | Relative humidity | Atmosphere: *RH*_atm_  Surface: *RH*_surface_ | % |
| $h$ | Convective and conductive coefficient | Calculated from Nusselt number | [W m^-2^ K^-1^] |
| $P_{H_{2}O}$ | Saturated water vapor pressure | a = 8.07131;  b = 1730.63;  c = 233.426 | [Pa] |
| $P_{\infty}$ | Atmospheric pressure | $1.01\times10^{5}$ | [Pa] |
| $M$ | Relative molecular mass | $\begin{matrix} M_{H_{2}O}:18 \\ M_{air}:29 \end{matrix}$ | Unitless |
| $c_{p}$ | Specific heat capacity of air at constant pressure | $1.012\times10^{3}$ | [J Kg^-1^ K^-1^] |
| $Le$ | Lewis number | 0.87 | Unitless |
| *n* | Power coefficient of *Le* | Natural convection (horizontal or inclined plate): -3/4 | Unitless |


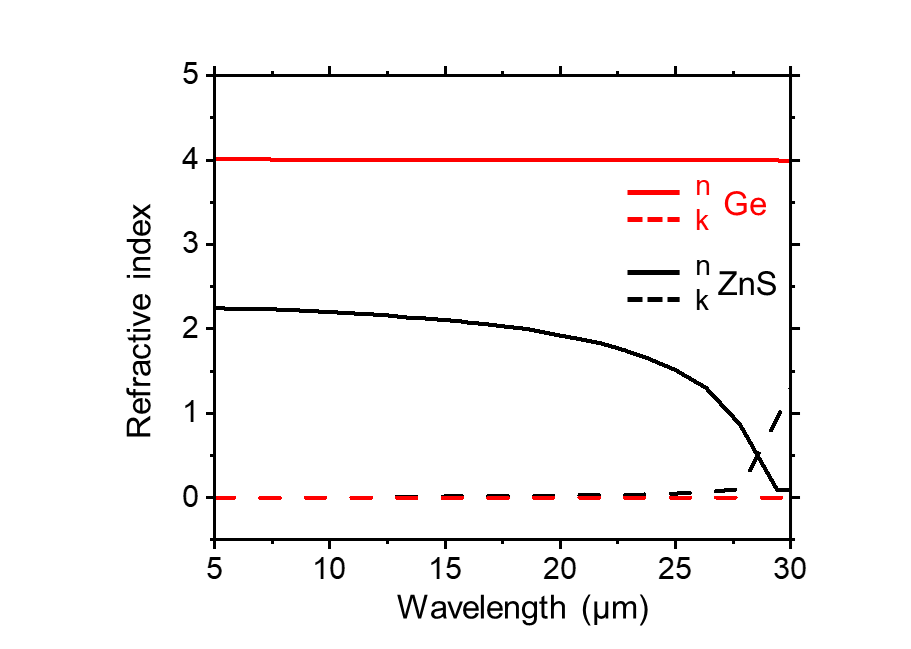


**Fig. S1** Refractive indexes of Ge (red) and ZnS (black).


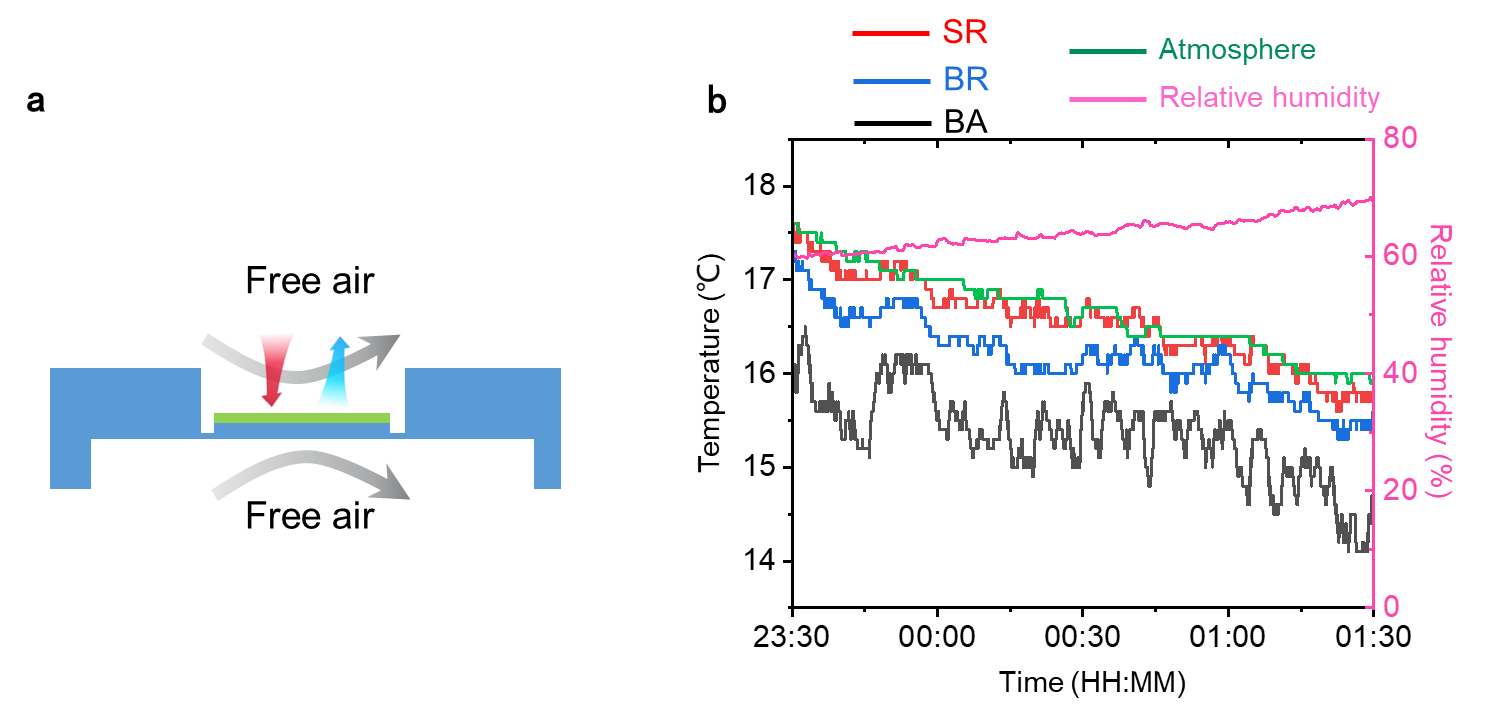


**Fig. S2 Thermal test with direct exposure to air.** (a) Schematic diagram of the experiment setup. The wind shield and the bottom insulating foam are removed to mimic the real environment. (b) Real-time temperature of selective reflector (red), broadband reflector (blue), broadband absorber (black), and atmosphere (green).


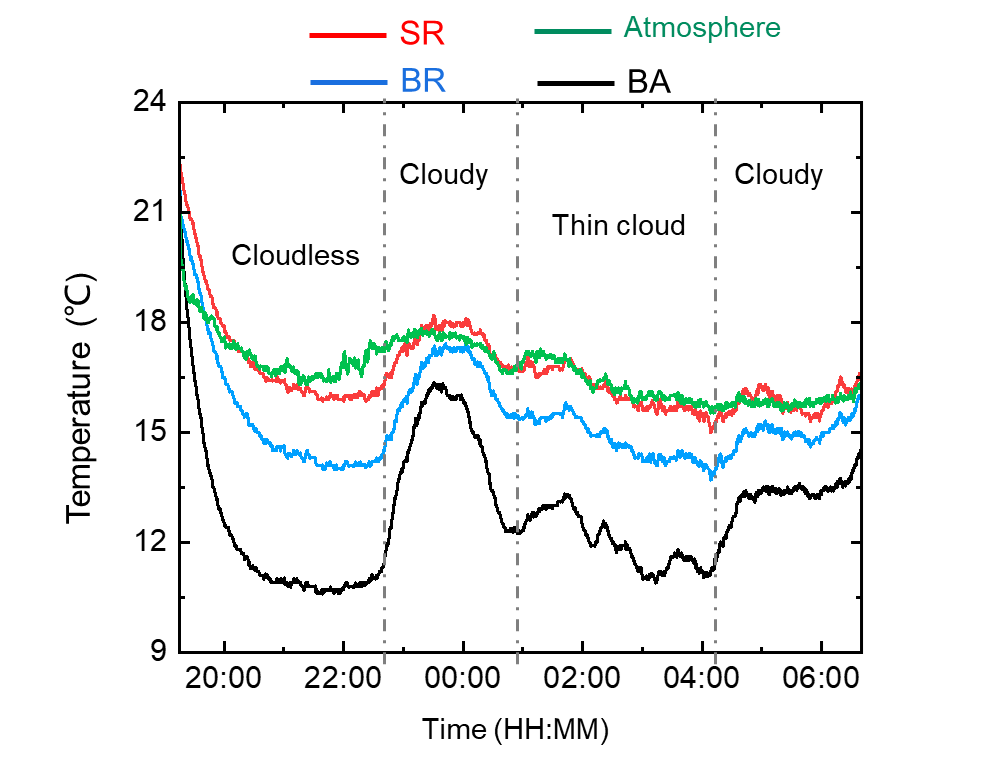


**Fig. S3 Thermal test on a cloudy night.** The temperature of the three samples all exhibit a temperature rise when there are clouds, while the temperature of SR is always higher than that of BR and BA. During the cloudy time around 0 a.m., the temperature of SR is even higher than the atmosphere temperature, which may due to non-uniformity of atmospheric temperature in the longitudinal direction.

**Supplement 2 Calculation of annual energy-saving for anti-condensation**

The hourly atmospheric temperature and relative humidity data of each city over the year of 2022 are downloaded from the website: <https://rp5.ru/Weather_in_the_world>. Only the night-time is taken into account since the heat transfer analysis in this work consider only when solar irradiance is absent.

The calculation is conducted by comparing the minimum active heating power required to maintain the temperature above the dew point with and without the selective reflector, which can be written as

$P_{save}=\sum_{t_{i}\in2022} (P_{w/o\_SR}-P_{w/\_SR})_{t_{i}}$ (S8)

Take the situation that without SR for example, the energy balance at the surface without SR at a certain time $t_{i}$ can be written as

$\alpha_{1}(T_{dew})P_{1}+(1-r_{2}(T_{dew}))P_{2}-P_{\text{conv,cond}}-P_{w/o\_SR}=0$ (S9)

where $T_{dew}$ (K) is the dew point which can be calculated by:

$T_{dew}=\frac{243.12*[\log(RH)+17.62*T_{atm}/(243.12+T_{atm})]}{17.62-[\log(RH)+17.62*T_{atm}/(243.12+T_{atm})]}$ (S10)

Solving equation (S9), we can get hourly active heating power required to maintain the temperature above the dew point. The final summing process will remove all negative terms, since no extra heating power is needed to prevent condensation at that certain time.

$\left(P_{w/o\_SR}\right)_{t_{i}}=0,if\left(P_{w/o\_SR}\right)_{t_{i}}<0$ (S11)


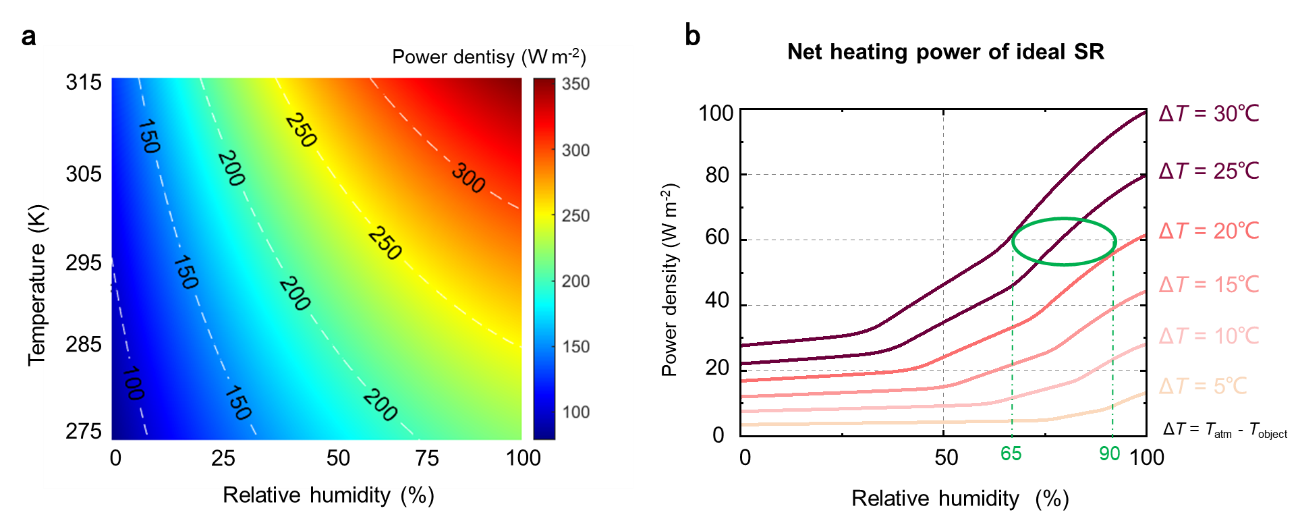


**Fig. S4** (a) Calculated atmospheric radiation power as a function of temperature and humidity. (b) Net heat power of ideal selective reflector in different environments. The atmospheric radiation intensity and transmittance vary with humidity, and the net heating power increases with the humidity. In extreme environment, when the temperature of the object is much lower than that of the atmosphere and the humidity is high, the net heating power can reach to 60 W m^-2^.

**
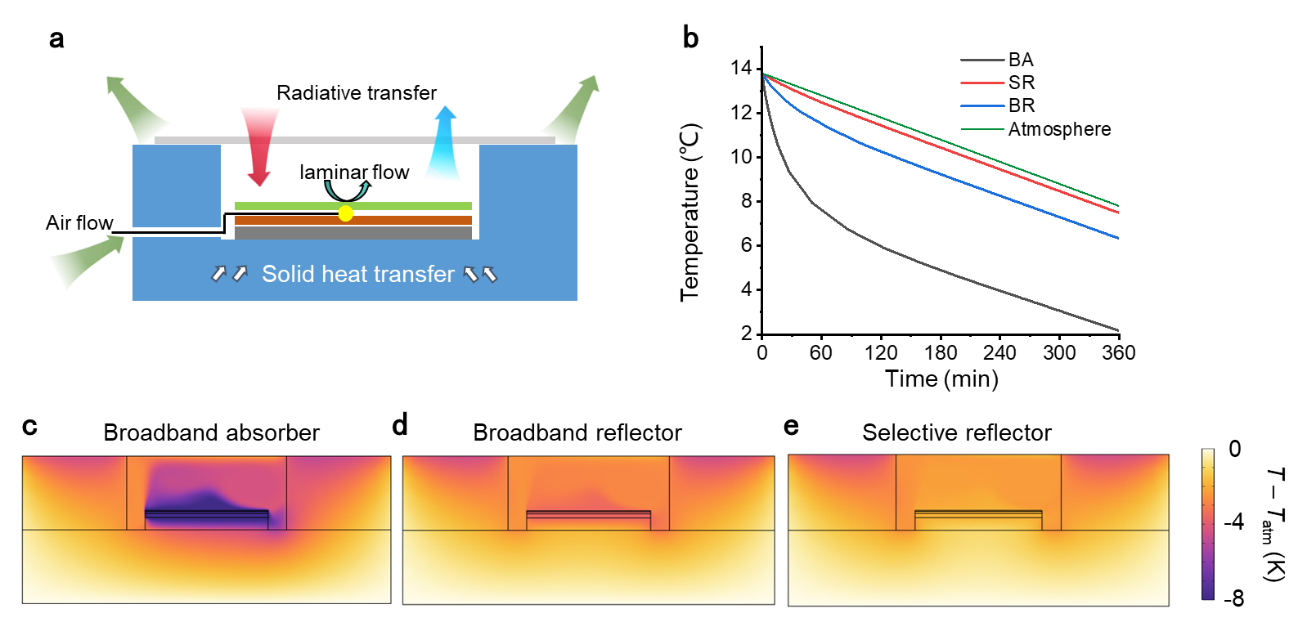
**

**Fig. S5 Thermal analysis of the experimental setup.** (a) Schematic illustration of the two-dimensional model. Here, the solid heat transfer in the box and the samples, the natural laminar flow inside the chamber, the radiative transfer of the surfaces, and the convective heat transfer caused by the unconfined holes are all considered in the simulation. (b) Simulated time-dependent temperature of different emitters. The atmosphere temperature in simulation is a linear fitting of experimental data. (c-e) Simulated temperature distribution (T_surf_ – T_atm_) in the experimental setup of (c) broadband absorber, (d) broadband reflector, and (e) selective reflector.
